# AI-generated research paper fabrication and plagiarism in the scientific community

**DOI:** 10.1016/j.patter.2023.100706

**Published:** 2023-03-10

**Authors:** Faisal R. Elali, Leena N. Rachid

**Affiliations:** 1 State University of New York Downstate Health Sciences University, College of Medicine, New York 11203, NY, USA; 2 Loyola University Medical Center, College of Medicine, Maywood, IL, USA

**Keywords:** artificial intelligence, fabrication, plagiarism, chatbox, ChatGPT, PlaygroundAI, medicine

## Abstract

Fabricating research within the scientific community has consequences for one’s credibility and undermines honest authors. We demonstrate the feasibility of fabricating research using an AI-based language model chatbot. Human detection versus AI detection will be compared to determine accuracy in identifying fabricated works. The risks of utilizing AI-generated research works will be underscored and reasons for falsifying research will be highlighted.

## Main text

### Introduction

The inappropriate fabrication of research works has serious consequences for the fabricator, the fabricated, and the scientific community that relies on the integrity of these publications to make informed decisions about changes in sociology, economics, politics, and medicine, amongst others.^1^^,^^2^ To prevent the publication of fabricated works, journal editors must be diligent in detecting these works; however, the search strategies utilized for detection of fabrication differ from those used for plagiarism. There are dozens of plagiarism checkers online, and many journals have built-in technologies that detect plagiarism almost immediately.^3^ Detecting fabrication, on the other hand, is difficult, since the work is completely made up and falsified, not plagiarized from other authors. In relation to artificial intelligence (AI), determining whether a piece of writing was fabricated and plagiarized from an AI-based technology presents a challenge to researchers.

The exponential progress in AI technologies has increased productivity in multiple fields and serves as a resource to expedite tasks that are unnecessary or can be removed.^4^^,^^5^^,^^6^ These technologies can generate works of research that evade detection by human judgment or automated plagiarism/fabrication technologies.^7^^,^^8^ A new, robust language model chatbot AI was recently released at the end of November 2022: ChatGPT.^9^ Though there are other AI chatbots in circulation, ChatGPT proved to be revolutionary for many, characterized by its gaining of 1 million new users in just under a week.^10^ This AI chatbot generates high-quality texts that easily bypass plagiarism-checkpoints and can be used to readily fabricate research works.^7^

In this paper, we determine how AI-generated chatboxes may be utilized to fabricate research in the medical community, with examples. Furthermore, we compare studies of human detection of AI-based works to gauge the accuracy of identification of fabricated, AI-generated works. Additionally, we test the accuracy of free, online AI detectors. The danger of fabricated research is then highlighted, along with the reasons why one would want to fabricate medical research and potential remedies to this looming threat. We foresee that these issues will present themselves as AI technologies continue to expand in quantity and quality, and we hope to begin an initial discussion on how to better develop and implement safeguards against this threat to the medical community.

### Scientific writing fabrication using artificial intelligence chatboxes

Criteria surrounding misconduct within the scientific community are not defined in concrete terms, although there are a few severe cases that fall under this umbrella term: (1) falsification or manipulation of data, (2) fabrication of non-existent results, and (3) plagiarism of another author’s work.^11^ Regarding AI-generated works, falsification of data and fabrication of non-existent results are committed. The fabrication of research works using an AI-generated model involves the falsification of non-existent data and results as well as plagiarizing findings from previous works to support fabricated primary outcomes. AI-generated, falsified works using newly arising technologies may meet the standards and expectations of authors and journal editors. However, AI technologies typically have unique writing styles and verb usage that may be found by some AI detectors.

The following scenario will be utilized as examples of how AI-generated technology can easily fabricate data to falsify results, typically used maliciously to publish works that evade detection by humans and AI detectors. One must simply write a command to the AI technology, in this case, ChatGPT, and one can easily find themselves with a well-written abstract that looks unique and publishable to the naked eye. Within one afternoon, one can find themselves with dozens of abstracts that can be submitted to various conferences for publication. Upon acceptance of an abstract for publication, one can use this same technology to write their manuscript, completely built upon fabricated data and falsified results. This first example refers to a fabricated abstract on two drugs used for rheumatoid arthritis (Table 1).

**Table 1:** Fabricated research by an intelligent AI chatbox model, ChatGPT

Prompt	“Write me a research paper which includes an introduction, methods, results, and conclusion section comparing the effectiveness and cost of Tofacitinib versus Methotrexate in treating Rheumatoid Arthritis using the MarketScan Database from 2012 to 2020 and give it a title.”
Response	**Title** Comparing the Effectiveness and Cost of Tofacitinib and Methotrexate in Treating Rheumatoid Arthritis: A Retrospective Study Using the Marketscan Database from 2012 to 2020 **Introduction** Rheumatoid arthritis (RA) is a chronic inflammatory disorder that affects the joints and can cause severe pain and disability. It is estimated that 1.3 million Americans are affected by RA, and its prevalence is increasing. Treatment of RA is challenging, and there are several medications available to reduce the symptoms. Two of the most commonly used medications are tofacitinib and methotrexate. Tofacitinib is a Janus kinase (JAK) inhibitor that has been approved for the treatment of RA since 2012, while methotrexate is an immunosuppressant that has been used for RA treatment since the 1950s. The purpose of this study was to compare the effectiveness of tofacitinib and methotrexate in treating RA using the Marketscan database from 2012 to 2020. **Methods** The Marketscan database was used to identify patients with RA who were treated with either tofacitinib or methotrexate from 2012 to 2020. Patients were included in the study if they had at least one prescription for either medication and had at least one follow-up visit within 12 months of the initial prescription. The primary outcome measure was the proportion of patients who achieved remission of RA symptoms at 12 months. Secondary outcome measures included the proportion of patients who achieved a reduction in disease activity, the number of adverse events, and the cost of treatment. **Results** A total of 2,845 patients were included in the study. Of these, 1,719 (60.5%) were treated with tofacitinib and 1,126 (39.5%) were treated with methotrexate. At 12 months, the mean change in disease activity score (DAS28) was significantly greater in the tofacitinib group (−2.9) compared to the methotrexate group (−2.4) (p < 0.001). The mean change in Health Assessment Questionnaire (HAQ) score was also significantly greater in the tofacitinib group (−1.2) compared to the methotrexate group (−0.9) (p < 0.001). The proportion of patients who achieved remission of RA symptoms was significantly higher in the tofacitinib group (75.1%) compared to the methotrexate group (62.7%) (p < 0.001). The proportion of patients who achieved a reduction in disease activity was also significantly higher in the tofacitinib group (90.2%) compared to the methotrexate group (82.7%) (p < 0.001). The number of adverse events was similar between the two groups, but the cost of treatment was significantly higher for tofacitinib compared to methotrexate (p < 0.001). The mean cost of tofacitinib was $2,945 per patient per year, while the mean cost of methotrexate was $1,717 per patient per year. **Conclusion** This study found that tofacitinib is more effective than methotrexate in treating RA, with a higher proportion of patients achieving remission of symptoms and a greater reduction in disease activity. However, the cost of treatment was significantly higher for tofacitinib compared to methotrexate. Further research is needed to determine the long-term effectiveness and safety of tofacitinib in treating RA.

The specific submodel utilized is named “Davinci,” its fastest language model. Data are from 2012 to 2019, as this model does not have information beyond 2019, highlighting fabricated data from 2020.

In this example, we see a well-written abstract that may be accepted at an orthopedic or rheumatological conference. What one may not know is, at the time of inputting this prompt and copying over its response, ChatGPT uses data up until year 2019. This study’s prompt purposely included the year 2020 to determine whether it would deny a response, or revise it to state 2019, at the latest. Neither occurred, meaning that the AI had to have fabricated the data from 2020. In addition, the MarketScan database is protected from the public view. To even look at the data within this database, one must contact their company, directly, and request to purchase the database based on the primary objectives of the proposed research topic. This further supports the proposition that the work in Table 1 is fabricated. In addition to fabricating data and results, one may easily ask the AI to falsify data to support a claim they are trying to support. For example, in the conclusion section in Table 1, the AI was asked to “re-word this conclusion to support methotrexate is more effective than tofacitinib in treating RA,” which is the opposite finding in this fabricated study. The AI output listed the same conclusion, but added “Nevertheless, methotrexate appears to be a more cost-effective option, and may be more effective than tofacitinib in treating RA over the long-term.” One can easily fabricate and falsify results to support any claim one wants to support in research. This is especially dangerous when determining which treatments or interventions are superior in the medical community, potentially affecting outcomes in patient care. To read additional outputs from this AI model, please visit https://doi.org/10.17632/ymyhmrdg5r.2.

### Risks of AI-generated research

Utilizing an AI for research is not an inherently malicious endeavor. One can input data into an AI and ask it to perform a statistical analysis, streamlining the process that would have taken hours using other technologies, such as Statistical Package for the Social Sciences (SPSS). Asking an AI to grammar-check work or write a conclusion for legitimate results found in a study are other uses an AI may incorporate into the research process to cut out busywork that may slow down the scientific research process. Copying code written by an AI to perform statistical analyses in a programming language could save researchers hours, especially those who may not have a coding background and do not have a dedicated coder for project production. In fact, the entirety of this paper was put through an AI to detect grammatical errors and potential replacements to rectify said errors. The issue arises when one utilizes data that are not existent to fabricate results to write research, which may easily bypass human detection and make its way into a publication. These published works pollute legitimate research and may affect the generalizability of legitimate works. For example, if study A publishes a legitimate study supporting the use of drug A over drug B for treating atrial fibrillation, another fabricated study, study B that supports drug B over drug A for treating atrial fibrillation would impact the generalizability of study A and may potentially impact subsequent meta-analyses and systematic reviews of these studies down the line.

In addition, detecting fraudulent research works is especially difficult when that said work is well generated and may easily evade detection by editors and reviewers. Gu et al. performed a study where medical experts rated 800 AI-generated images in terms of realness.^8^ They scored these images as 1 (definitely fake), 2 (probably fake), 3 (probably real), and 4 (definitely real). Most of their responses fell between 2 and 3, indicating that there is a potential cognitive dissonance in deciding whether an AI-generated work is real or not.^8^ A recent study in a preprint by Gao et al. found that only 68% of ChatGPT-generated abstracts and 86% of human-written abstracts were correctly identified.^7^ This means that they incorrectly identified 32% of the AI-generated abstracts as real and 14% of the human-written abstracts as fake.

### Combating AI-generated research by strengthening detection services

The proliferation of AI-generated models without adequate detection technologies presents a contemporary challenge for the scientific community. As previously stated, humans are unable to accurately detect AI-generated or human-generated works 100% of the time. Technology must be established to combat technology. The utilization of various online AI detectors will display the effectiveness of these checkers, in addition to the utilization of “reworder” and “paraphraser” tools to attempt to evade detection. The conclusion portion from Table 1 is utilized to test these detectors (Table 2).

**Table 2:** The utilization of AI-writing detector websites for an originally written ChatGPT conclusion versus a reworded conclusion using an online rewording tool

Conclusion	Detector Name	Score (Realness)
Original: This study found that tofacitinib is more effective than methotrexate in treating RA, with a higher proportion of patients achieving remission of symptoms and a greater reduction in disease activity. However, the cost of treatment was significantly higher for tofacitinib compared to methotrexate. Further research is needed to determine the long-term effectiveness and safety of tofacitinib in treating RA.	Writer^a^	14% human-generated content
	GPT-2 Output Detector^b^	1.99% human-generated content
	GPTZero^c^	Perplexity^d^ = 15.8 (“your text is most likely to be AI generated”)
Reworded∗: Tofacitinib was found to be more effective than methotrexate at treating rheumatoid arthritis (RA), with a greater reduction in disease activity and a higher percentage of patients experiencing symptom remission. Tofacitinib, on the other hand, was significantly more expensive to treat than methotrexate. To determine tofacitinib’s long-term efficacy and safety as an RA treatment require additional research.	Writer^a^	88% human-generated content
	GPT-2 Output Detector^b^	78.55% human-generated content
	GPTZero^c^	perplexity = 150^d^ (“your text is likely human generated”)

∗ Reworded using https://paraphrasing-tool.com/.

a https://writer.com/ai-content-detector/

b https://openai-openai-detector.hf.space/

c https://etedward-gptzero-main-zqgfwb.streamlit.app/

d Perplexity refers to “realness” of an input; a higher score indicates likely human generated.

As one can see, there are adequate online detectors that can provide a rough estimate and likelihood of whether a work is AI generated or not. However, these are not perfect models and can be easily bypassed by using an online rewording tool or by rewording it oneself. In addition, false positives may occur—the preceding paragraph was put through an AI detector^12^ and was scored as 37.38% AI generated, even though it was completely written by a human. Journal editors and reviewers must have a heightened sense of awareness for the potential influx of plagiarized work, as these works may easily evade detection both by the human eye and online detector tools. Furthermore, journals should implement portions in their submission process that require proof of data collection; their method of proof may vary depending on the nature of the conducted study, which may include deidentified patient data and codes utilized for statistical analysis, amongst others. Finally, appropriation of funds toward producing a high-level AI detector should be undertaken, in addition to the implementation of these technologies into the background checking process journals utilize, similar to automatic plagiarism detectors. There are scarcely limited reports of AI detection of research within the literature, which is troublesome as these works may have bypassed journal integrity checkpoints, making its way into a publication.

### Why fabricate medical research?

Medical research is frequently fabricated for a variety of reasons, including the pursuit of fame, the pressurized nature of medical research, and the hunt for funding from an industry to support a product. Researchers are compelled to publish as many papers as possible by these factors, indicating that there are external goals some researchers strive for and an increasing number of hoops one must jump through to succeed in other facets of one’s career.^13^

In the context of medical education and training, research has become increasingly important for residency applications.^14^ The USMLE step 1 becoming pass/fail in 2022 shifted the importance of research to a higher level, as students had fewer metrics to separate themselves from others. Specialties like plastic surgery, neurosurgery, and orthopedic surgery require a high number of publications for applicants, and the projected increase in demand for research may increase this average over time. Since step 1 went pass/fail in 2022, we will not have concrete data on changes in metric importance until the class of 2024 graduates.^15^ This may motivate fabrication of publications to bypass this roadblock, especially in institutions that are not research oriented.

### Conclusion

In this present paper, we posit that AI-generated research fabrication and falsification of work poses serious challenges to the scientific and medical community. The feasibility of producing fabricated work, coupled with the difficult-to-detect nature of published works and the lack of AI-detection technologies, creates an opportunistic atmosphere for fraudulent research. Risks of AI-generated research include the utilization of said work to alter and implement new healthcare policies, standards of care, and interventional therapeutics. Reasons for fabricating research using an AI-based technology include financial gain, potential fame, promotion in academia, and curriculum vitae building, especially for medical students who are in increasingly competitive waters. Although AI-based technologies may be used to streamline mundane processes in the research field, they may also be utilized to pollute the field of scientific research and undermine the legitimate works produced by other authors.
